# Prediction of emergence from prolonged disorders of consciousness from measures within the UK rehabilitation outcomes collaborative database: a multicentre analysis using machine learning

**DOI:** 10.1080/09638288.2022.2114017

**Published:** 2022-08-27

**Authors:** Richard J. Siegert, Ajit Narayanan, Lynne Turner-Stokes

**Affiliations:** a School of Clinical Sciences, Auckland University of Technology, Auckland, New Zealand; b School of Engineering, Computer and Mathematical Sciences, Auckland University of Technology, Auckland, New Zealand; c Department of Palliative Care, Policy and Rehabilitation, Faculty of Life Sciences and Medicine, King’s College London, London, UK; d Regional Hyper-acute Rehabilitation Unit, Northwick Park Hospital, London North West University Healthcare NHS Trust, London, UK

**Keywords:** Prolonged disorders of consciousness, PDOC, vegetative or minimally conscious states, prediction, outcomes, logistic regression, artificial neural networks, machine learning

## Abstract

**Purpose:**

Predicting emergence from prolonged disorders of consciousness (PDOC) is important for planning care and treatment. We used machine learning to examine which variables from routine clinical data on admission to specialist rehabilitation units best predict emergence by discharge.

**Materials and methods:**

A multicentre national cohort analysis of prospectively collected clinical data from the UK Rehabilitation Outcomes (UKROC) database 2010–2018. Patients (*n* = 1170) were operationally defined as “still in PDOC” or “emerged” by their total UK Functional Assessment Measure (FIM + FAM) discharge score. Variables included: Age, aetiology, length of stay, time since onset, and all items of the Neurological Impairment Scale, Rehabilitation Complexity Scale, Northwick Park Dependency Scale, and the Patient Categorisation Tool. After filtering, prediction of emergence was explored using four techniques: binary logistic regression, linear discriminant analysis, artificial neural networks, and rule induction.

**Results:**

Triangulation through these techniques consistently identified characteristics associated with emergence from PDOC. *More* severe motor impairment, complex disability, medical and behavioural instability, and anoxic aetiology were predictive of non-emergence, whereas those with *less* severe motor impairment, agitated behaviour and complex disability were predictive of emergence.

**Conclusions:**

This initial exploration demonstrates the potential opportunities to enhance prediction of outcome using machine learning techniques to explore routinely collected clinical data.

**Implications for rehabilitation**
- Predicting emergence from prolonged disorders of consciousness is important for planning care and treatment.
- Few evidence-based criteria exist for aiding clinical decision-making and existing criteria are mostly based upon acute admission data.
- Whilst acknowledging the limitations of using proxy data for diagnosis of emergence, this study suggests that key items from the UKROC dataset, routinely collected on admission to specialist rehabilitation some months post injury, may help to predict those patients who are more (or less) likely to regain consciousness.
- Machine learning can help to enhance our understanding of the best predictors of outcome and thus assist with clinical decision-making in PDOC.

## Introduction

Enhanced emergency services (e.g., helicopter evacuation, acute major trauma centres, defibrillators in public places) have substantially improved the outcomes for many survivors of medical emergency. However, as we get even better at saving lives, a proportion of patents who would otherwise have died at the scene of injury now survive with catastrophic brain injury and are left in a prolonged disorder of consciousness (PDOC—i.e., vegetative or minimally conscious state)—sometimes permanently [1].

In the early stages post injury, it is appropriate to treat patients expectantly in the hope of a good recovery, but the longer they remain in PDOC the less likely they are to emerge into consciousness. Better prognostic information may help to guide clinicians and patient’s families about an individual’s expectations for recovery and inform decisions about care and treatment. Unfortunately, there is a dearth of information on which to base such predictions. Factors that are known to be associated with more favourable outcomes include age, severity, type of injury (trauma vs non-trauma), and level of consciousness [2–4], but as yet there is no recognised algorithm for combining these to predict individual outcomes with any degree of accuracy. Successive studies have repeatedly identified the same factors, not necessarily because they are the strongest predictors, but because they happen to be information that is readily available in acute settings [5–7]. When patients leave the acute hospital and transfer to rehabilitation, a range of other measures are recorded and it is pertinent to consider whether any of these may assist with more accurate predictions about which patients may emerge into consciousness.

In the UK, assessment and management of prolonged disorders of consciousness is primarily provided in designated specialist rehabilitation units. The UK Rehabilitation Outcomes Collaborative (UKROC) [8] provides the national clinical database for all specialist in-patient rehabilitation services in England. Although a PDOC registry is in development, as yet the dataset does not include tools formally designed to assess the level of consciousness. However, it does include a rich dataset of patient-level information that includes measures of needs, inputs, and outcomes from rehabilitation. In the US, the Traumatic Brain Injury Model Systems (TBIMS) national database provides the largest longitudinal dataset in the world for monitoring outcomes [9]. Analyses of this dataset have used the lowest possible total score on the Functional Independence Measure (FIM = 18) [10] as a proxy for identifying patients in VS [11]. The UKROC dataset routinely collects the UK Functional Assessment Measure (UK FIM + FAM) [12] (an extended version of the FIM) as its primary outcome measure. This provides an opportunity to take a similar approach with the UK national dataset, using modern statistical techniques such as machine learning.

Traditional analytical approaches may use logistic regression techniques centred on hypothesis-based algorithms to identify characteristics in the data that may predict emergence from PDOC (either individually or in combination), but other methods can also be used. Machine learning (ML) involves the application of algorithms and techniques for generating statistical and rule-based models from data using patterns and inference without necessarily testing specific hypotheses. It involves techniques for finding patterns in data using algorithms (e.g., neural networks, decision trees, clustering, random forest) which can provide insights and enhance fast and accurate decision-making [13]. In particular, such models can be useful for generating explanations and predictions through identification of influential variables. ML can be *supervised* where the object is to predict a specified outcome (e.g., alive/dead) or *unsupervised* where the outcomes are not preordained and a technique such as clustering might be used to determine the outcome categories [14].

ML algorithms (MLAs) have been successfully applied in marketing and finance for some time, but their use in the health and social sciences has only become widespread recently [14]. This is changing quickly, however, and their application has important implications for diagnosis, prognosis and treatment [15]. For example, Chekroud et al. developed an MLA that showed promise for predicting which patients responded to one antidepressant rather than another [16]. In rehabilitation studies, MLAs have been used for guiding planning for home care clients, predicting risk of acute care readmission among rehabilitation inpatients and predicting functional status of community-dwelling older people 12 months later [17–19]. Other applications of ML in rehabilitation have included: decision tree models to predict ventilator associated pneumonia after traumatic brain injury (TBI), accelerometer-based algorithms to classify physical activity after acquired brain injury (ABI), and prediction of outcomes after hip fracture [20–22].

For a comprehensive and accessible introduction to the application of ML approaches in health research we recommend the review by Dwyer et al. [14]. MLAs including “neural networks” (or more correctly speaking *artificial* neural networks) have some advantages over traditional statistical methods. First, they do not rely upon any assumptions about the data conforming to any particular statistical distribution. Second, they typically involve “training” the network so that its ability to predict specific outcomes on withheld data can improve over repeated presentations of different subsets (partitions) of data. Third, MLAs have the potential to identify variables that are predictors of an outcome that researchers might not have even considered, thus generating novel hypotheses. However, the reasons for their predictive and classification behaviour are not always clear when neural networks are used, because they use mathematical weights to combine variable values and functions that transform or “squash” combined values into class-based output. In other words the neural network can be “trained” to be highly accurate at predicting the outcome of interest but the researcher is unable to specify the precise algorithm underpinning its classification. For that reason, researchers frequently combine neural networks with more informative MLA approaches, such as “rule induction” methods, e.g., decision trees, to help them identify possible reasons for model behaviour and specific thresholds for use in antecedents of rules for prediction.

In this first feasibility study, we apply two machine learning techniques to examine the extent to which data items contained within the UKROC clinical dataset might contribute to the prediction of which patients admitted in PDOC may emerge into consciousness by the end of the programme, and how these techniques might enhance the predictions derived from traditional logistic regression and linear discriminant analyses.

## Materials and methods

### Design

A large multicentre national cohort analysis of prospectively collected clinical data from the UK Rehabilitation Outcomes Collaborative (UKROC) national clinical database 2010–2018.

#### Setting and participants

In England, the majority of patients with mild-moderate brain injury receive rehabilitation within their local non-specialist Level 3 services. Those with more complex rehabilitation needs are referred to Level 1 (regional) to Level 2 (district) specialist rehabilitation services. The programmes provided within the specialist services are set out in the NHS England service specification [23] and include assessment and management of prolonged disorders of consciousness.

Participants were all adults (aged 16-plus) who were admitted to a Level 1 or 2 specialist rehabilitation service in England during the eight-year study period who were in (or likely to be in) a prolonged disorder of consciousness on admission.

#### Data source—the UKROC database

The UKROC database was established in 2009 through funding a programme grant from the UK National Institute for Health Research (NIHR) [24] but now provides the national commissioning dataset for NHS England. Completed rehabilitation episodes are collected by each provider on local dedicated software and are uploaded at monthly intervals to a secured NHS server held at Northwick Park Hospital, London.

The dataset comprises socio-demographic and process data (waiting times, discharge destination, etc.) as well as clinical information on rehabilitation needs, inputs and outcomes. Full details may be found on the UKROC website: http://www.csi.kcl.ac.uk/ukroc.html.

The data reporting requirements for Level 1 and 2 services have evolved over time and vary somewhat between the different levels of service. Systematic data collection started in April 2010, but reporting of the full dataset was initially voluntary. Since April 2013, services commissioned centrally by NHS England are required to report the full UKROC dataset for all admitted episodes [25], but some locally commissioned Level 2 services still report only a reduced dataset. All units registered with UKROC receive training in use of the tools to support accurate data collection, and have access to update workshops and telephone support.

### Measurement tools

Specific tools within the dataset include the following measures:

- **The Neurological Impairment Set (NIS**) [26] records the severity of neurological (e.g., motor, sensory, cognitive, communicative and psychological) impairments.
- **The Patient Categorisation Tool (PCAT**) [27,28] records the types of need a patient may have that lead to a requirement for treatment in a specialist rehabilitation unit.
- **The Rehabilitation Complexity Scale (RCS-E)** [29,30] is an ordinal scale that records the resource requirements to meet the patient’s needs for medical support, basic care and nursing, therapy, and equipment.
- **The Northwick Park Dependency Score (NPDS)** [29,31] is an ordinal scale of basic care and nursing dependency on nursing staff time (number of helpers and time taken to assist with each task) designed to assess needs for care and nursing in clinical rehabilitation settings [31]. It is shown to be a valid and reliable measure of needs for care and nursing in rehabilitation settings [32].
- **The UK Functional Assessment Measure (UK FIM + FAM)** [33] is a global measure of disability comprising 16 items addressing physical function (FIM + FAM motor) and 14 addressing cognitive, communicative and psychosocial function (FIM + FAM cognitive). Each item is scored on a seven-point ordinal scale from 1 (total dependence) to 7 (complete independence) giving a total score range of 30–210. Further details are published elsewhere [12,33]. Collected on admission and discharge the UK FIM + FAM forms the principal measure of outcome (change in physical and cognitive disability) within the UKROC dataset.

#### Identification of patients in prolonged disorders of consciousness

As noted above, the FIM + FAM does not provide a direct measure of consciousness; however, a recent study has demonstrated that, at large population level, it can provide a reasonable proxy. In 312 patients admitted to a designated specialist PDOC assessment and management programme, UK FIM + FAM scores were examined alongside formal detailed evaluation of consciousness including validated measures (e.g., the Coma Recovery Scale (CRS-R) [34] and the Wessex Head Injury Matrix (WHIM) [35]). Examination of the area under the ROC curve demonstrated that total UKFIM + FAM scores of < =31 and > =36 would, respectively, provide a reasonably robust separation of low level PDOC (VS/MCS-minus) versus emerged into Consciousness for the purpose of future evaluations [36]. For the purpose of this study, scores of 32–35 were excluded to provide clear separation and reduce the chance of cross contamination.

### Data extraction

De-identified data were extracted for all recorded in-patient episodes for adults aged 16+ admitted to a Level 1 or 2 specialist rehabilitation service and discharged during the eight-year period between April 2010 and July 2018, if they had:

- An acquired brain injury with total FIM + FAM score on admission ≤31 (“PDOC on admission”).
- A length of stay 8–400 days (i.e., plausible admissions for PDOC assessment management).
- Valid ratings of the RCS-E (version 12), PCAT and NPDS on admission, and of the UK FIM + FAM on both admission and discharge.

This resulted in 1170 patients admitted to a total of 53 centres in England.

### Analyses

Four different options for statistical analyses were explored:

- Binary logistic regression (LR) constructs linear combinations of one or more independent variables to model a binary dependent variable, typically labelled “0” and “1.” LR makes no assumptions about the distribution of the independent variables. Instead, odds ratios for each independent variable are estimated through calculating the ratios of being in one dependent class as opposed to the other class and then converting these log-odds into probabilities and regression coefficients. Variables can be entered together (block entry) or stepwise. Conditional stepwise entry was used in the analysis below. Binary LR is used mainly for data-fitting purposes and for identifying significant input variables for further model evaluation and classification.
- Linear discriminant analysis (LDA) produces linear classifiers for separating two or more classes of samples. Whereas LR finds the best fitting model through log-odds ratios and regression coefficients, LDA creates discriminant functions consisting of coefficients to maximise the difference between classes relative to the difference within the same class. Such coefficients can be interpreted as contributing to class assignment, where positive LDA coefficients indicate increases in standard deviations towards the higher valued class (emerged in our case, coded as “1” in the data) and negative as decreases towards the lower valued class (not emerged, “0”), with larger values signifying better predictors. Similar to LR, variables can be entered as a block or stepwise. LDA is typically used with cross-validation techniques to test the accuracy of classifier models (see below).
- Artificial neural networks for supervised ML use the concept of interlinked neurons (nodes) to perform general function approximations for learning a complex mapping from independent attributes to dependent attributes. In a three-layer perceptron neural network, independent attribute values are fed to the input layer of the perceptron, and mathematical weights on the links convert these input values to be new values at the hidden layer and the output layer using transfer functions that produce output from the weighted input. A threshold logic function, for instance, produces a 1 if a specific weighted sum threshold is exceeded, otherwise 0. A logistic sigmoid transfer function, on the other hand, produces output values between 0 and +1 according to the formula: $\sigma (x)=\frac{1}{1+e^{-x}},$ where σ(x) is the sigmoid value of the weighted sum x, and $e^{-x}$ is the natural logarithm of x. At the output level, a comparison is made between the actual output and the desired output to calculate an error sample by sample, and adjustments are made to the weights so that the error is reduced through gradient descent the next time the samples are presented. Each presentation of all samples is an epoch. The architecture chosen here consists of a layer of 12 input neurons (one for every attribute selected through filtering), a layer of 30 hidden neurons, and one output neuron for the emerged/not emerged state coded as 1 and 0, respectively, in the data. Parameters for learning include a learning rate (the maximum amount of weight change allowed per epoch, 0.2 in our case), momentum (the rate of convergence to an ideal set of weights, also set to 0.2), epochs (the number of presentations of all the data, set to 10 000) and termination condition (stop training if output error does not exceed 0.0001 in any epoch). Sensitivity analysis (how a trained neural network behaves under input and weight perturbations) is also used to identify which attributes influence output values the most.
- Rule induction algorithms typically generate decision trees for splitting samples using tests based on variable values so that samples of one class are separated from samples of another class in different branches of various tests. Chi-squared automatic interaction detector (CHAID) uses Pearson Chi-square *p* values to find the most significant variable for splitting samples at each level and each path of the tree until no more significant splits can be found. The tree is then converted into a rule set by tracing paths from the root node to every terminal node of the tree, with the antecedents of each rule consisting of branch tests and the consequent the class of samples at the terminal node. A probability value is associated with each rule regarding the rate at which that rule captures all samples in a specific class.

Models can be “data-fitting” or “cross-validated.” A data fit model uses all the data for model construction, with no samples withheld for testing. A cross-validated model uses only a subset of data for model construction and then tests the robustness of the model against the remaining withheld data. Common methods of cross-validation include leave-one-out (LOO) and *x*-fold. LOO constructs a model on all the samples except one and then tests the model on that withheld sample, repeated for every sample. This leads to the construction of as many models as there are samples, and the accuracy of prediction of LOO cross-validation (true positives plus true negatives over all samples) is reported at the end of all model evaluations. In *x*-fold cross-validation, the data is split into *x* equal-sized sets (typically, 10). A model is constructed on all but one set and then tested against the withheld set. This is repeated for every set with the overall average accuracy reported at the end of all evaluations.

For both data-fitting and cross-validation, area under receiver operating characteristic (AUROC) curves are used to display discrimination ability of the model by plotting the false positive rate (usually calculated as 1 minus specificity or the true negative rate) on the *x*-axis, and the sensitivity (the true positive rate) on the *y*-axis. The closer the AUROC figures to 1, the better the discrimination ability. In the experiments below, 0 (non-Emergence) is considered positive and 1 (Emergence) is negative, meaning that sensitivity refers to non-Emergence and specificity refers to Emergence. Below, regression is used for data-fitting only, and the three other approaches use both data-fitting and some form of cross-validation.

#### Our approach in this analysis

SPSS v26 was used for all data analysis.

The analysis proceeded in two stages: feature filtering, and model construction. Feature filtering methods remove the least interesting or relevant variables so that the remaining variables can be used for model construction. Filtering examines the influence of each variable separately on the grouping variable and does not look for combinations of variables. For the purpose of filtering, patients who scored ≥36 on the total FIM + FAM at discharge (“Emerged from PDOC”) were compared as a group against all other patients on a full range of admissions variables using bootstrapped T-tests. These admission variables included: Age, length of stay, time from onset to admission and all the individual items of the Neurological Impairment Scale (NIS), Rehabilitation Complexity Scale (RCS-E), Northwick Park Dependency Scale (NPDS) and the Patient Category Scale (PCAT). After filtering, those variables identified as significantly different between patients who remained in PDOC and those who emerged were entered into logistic regression models, a linear discriminant model, a neural network model and a rule induction model. For the purpose of model construction, the two discharge thresholds of PDOC ≤31 FIM + FAM and emerged ≥36 FIM + FAM were adopted, resulting in 1043 cases: 579 non-emerged from PDOC, 464 emerged from PDOC.

## Results

Table 1 shows the demographics and aetiology of the sample, broken down into those who did and did not emerge in consciousness by the end of the programme. Overall, just under half of the patients emerged into consciousness during the programme. Age and gender had little effect, the reason for the former may reflect the relatively young age of this sample (overall 48.4 years (SD 15.8). The overall mean time since onset was 231 days (95% CI 169, 321). It tended to be longer in those who remained in PDOC, but this did not reach significance due to wide confidence intervals. As expected, fewer patients with hypoxic injury emerged into consciousness. It should be noted that CVA patients who present in PDOC are those with profound brain injury, rather than typical stroke patients with more localised deficits. Nevertheless, the slightly higher rates of emergence may indicate that a proportion of them proved to be locked-in or had more localised deficits (e.g., Aphasia) that masked their initial potential for cognitive interaction.

**Table 1.** Breakdown of patients (*n* = 1043) on discharge, with age at admission broken down into four quartiles.

		At discharge from programme	
Parameter	Overall N	Still in PDOC (FIM + FAM< =31) n= (%)	Emerged (FIM + FAM> =36) n= (%)
Gender	*N* = 1043	*n* = 579 (54%)	*n* = 464 (46%)
Male	689	391 (68%)	298 (64%)
Female	353	187 (32%)	166 (36%)
Time since onset (days)			
Mean (95% CI)		297 (194, 468)	146 (94 232)
Aetiology diagnosis			
CVA^a^	309	141 (24%)	168 (36%)
Trauma	379	198 (34%)	181 (39%)
Hypoxia	241	187 (32%)	54 (12%)
Inflammatory	27	11 (2%)	16 (3%)
Tumour	18	12 (2%)	6 (1%)
Other^b^	57	27 (5%)	30 (7%)
Age at admission (Years)			
16–37	275	157 (27%)	118 (26%)
38–49	261	156 (27%)	105 (23%)
50–61	260	142 (26%)	118 (25%)
> =62	247	124 (22%)	132 (26%)
Mean (SD) years	1043	47.6 (15.8)	48.9 (15.9)

95% CI: 95% confidence interval.

^a^Cerebrovascular accidents due to infarct, haemorrhage, subarachnoid haemorrhage, or other aetiologies.

^b^Includes toxic/metabolic injuries e.g., hypoglycaemia, drug overdose, etc.

### Filtering

Initial filtering resulted in 12 significant variables for further analysis: “Eating,” “Drinking,” “Communication,” and “1:1 Specialling” items from the NPDS: “Neuro-psychiatric needs,” “Mood/emotion,” “Complex disability management,” “Tracheostomy/ventilator support,” “Behavioural,” and “Special equipment/Facilities” from the PCAT, “Medical” score from the RCS-E and the “Motor Subtotal” item from the NIS.

### Stage 1 logistic regression

The 12 variables were entered as scalar in a first logistic regression (LR) with entry probability 0.05 and removal 0.10, resulting in a significant data fit model (*p* ≤ 0.01) that explained between 18% (Cox and Snell R square) and 24% (Nagelkerke R square) of the variance using 6 of the 12 filtered variables: RCS-Medical, NIS-Motor, NPDS-Communication, PCAT-Behaviour, PCAT-Mood, and PCAT-Complex Disability. Data fit accuracy was 71% (84% sensitivity for “not Emerged from PDOC,” 54% specificity for “Emerged from PDOC”). Some improvement was obtained with a second LR where these six significant variables were entered as categorical for removal of non-significant values, resulting in a significant model (*p* ≤ 0.01) that explained between 28% and 38% of the variance. Data fit accuracy also improved to 77% (86% sensitivity, 66% specificity).

### Stage 2 linear discriminant analysis

LDA (stepwise entry at *F* = 0.05 and removal at *F* = 0.10) produced a single-function, six-variable model with 71% data fit accuracy (85% sensitivity for “not Emerged,” 53% specificity for “Emerged”), with Wilks’ Lambda of 0.81 (*p* ≤ 0.001). The six variables were the same as those found by logistic regression (Table 2). LOO stepwise LDA produced a similar 71% cross-validated accuracy model (84% sensitivity for “not Emerged,” 52% specificity for “Emerged”).

**Table 2.** The six variables found by LDA and their standardised canonical function coefficients.

Variables	Coefficients
NIS-Motor	0.40
NPDS-Communication	0.20
PCAT-Behaviour	−0.36
PCAT-Complex disability	0.33
PCAT-Mood	−0.24
RCS-Medical	0.31

NIS: Neurological Impairment Scale; NPDS: Northwick Park Dependency Scale; PCAT: Patient Categorisation Tool; RCS: Rehabilitation Complexity Scale.

**Table 3.** Independent variable importance for data-fitting ANN.

Independent variable on admission	Importance	Normalised importance
NIS-Motor	0.130	100.00%
NPDS-Communication	0.120	92.10%
NPDS-Eating	0.096	73.60%
PCAT-Behaviour	0.094	72.40%
PCAT-Psychiatric	0.094	72.40%
PCAT-Complex Disability	0.082	63.00%
RCS-Medical	0.076	58.00%
PCAT-Mood	0.067	51.50%
PCAT-Facilities	0.066	50.80%
NPDS-1:1 Specialling	0.062	47.50%
NPDS-Drinking	0.059	45.40%
PCAT-Tracheostomy	0.052	39.90%

NIS: Neurological Impairment Scale; NPDS: Northwick Park Dependency Scale; PCAT: Patient Categorisation Tool; RCS: Rehabilitation Complexity Scale.

### Stage 3 artificial neural network

**Figure 1.**
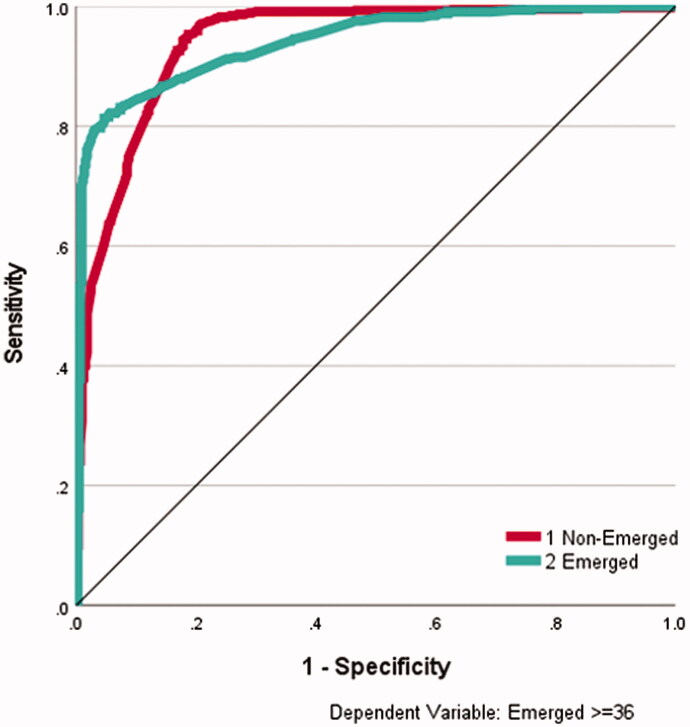
ROC curve for data-fitting artificial neural networks. ROC curve for data-fitting ANN for emerged (yes) and not emerged (no) The area under the curve was 0.94 for both emerged and non-emerged on discharge.

A 12-30-2 artificial neural network (ANN) perceptron architecture (12 input units, a layer of 30 hidden units and 2 output units, sigmoid transfer functions, gradient descent with 0.2 learning rate and 0.2 momentum, 10 000 epochs, training stopped if error does not exceed 0.0001 in any epoch) produced a data fit model of 89% accuracy (95% sensitivity for “not Emerged,” 82% specificity for “Emerged”). Variable sensitivity analysis revealed that NIS-Motor and NPDS-Communication were the most important variables (see Table 3) (100% and 93% normalised importance, respectively), followed by NPDS-Eating, PCAT-Behaviour and PCAT-Psychiatric (74%, 72%, and 72%, respectively). Figure 1 shows the AUROC curves for this data-fitting perceptron, with area figures of 0.94 for both the emerged and non-emerged class.

Using the same architecture for a 90% training/10% testing regime, repeated 10 times, produced an overall test accuracy of 69% (78% sensitivity for “not Emerged,” 57% specificity for “Emerged”), with NIS-Motor, NPDS-Communication, PCAT-Behaviour, and PCAT-Complex Disability regularly featuring as the most important variables (over 80% normalised importance), with NPDS-Eating also occasionally featuring above 70% importance.

### Stage 4 rule induction

Exhaustive CHAID generated a data fit six-rule set (two for “not Emerged,” four for “Emerged”) with an overall accuracy of 73% (78% sensitivity for “not Emerged,” 65% specificity for “Emerged”), as shown in Table 4.

**Table 4.** Rule induction—data fit six-rule set.

Rule	IF	THEN	Prediction Probability
1	NIS-Motor > 11 **AND** NIS-Moto*r* ≤ 13 **AND** PCAT-Behaviour ≤ 1 **AND** PCAT-Disability > 2	Not emerged	65%
2	NIS-Motor > 11 **AND** NIS-Moto*r* ≤ 13 **AND** PCAT-Behaviour > 1	Emerged	75%
3	NIS-Motor ≤ 11	Emerged	75%
4	NIS-Motor > 11 **AND** NIS-Moto*r* ≤ 13 **AND** PCAT-Behaviou*r* ≤ 1 **AND** PCAT-Disability ≤ 2	Emerged	55%
5	NIS-Motor > 13 **AND** PCAT-Complex Disability ≤ 2	Emerged	60%
When aetiology diagnosis subcategory on admission was included:			
6	NIS-Moto*r* > 13) **AND** PCAT-Complex Disabilit*y* > 2 **AND** (Diagnosis Subcategory = Anoxia **OR** (Stroke: Other) **OR** Toxic	Not emerged	91%

The best rule induced (91% capture) stated that non-emergence resulted when patients had an NIS-Motor admission score greater than 13, a PCAT-Complex Disability admission score >2 and diagnosis subcategory of Anoxia or Stroke or Toxic (Rule 6). Emergence, on the other hand, resulted from patients having an NIS-Motor admission score of 12 or 13, ≤1 on PCAT-Behaviour and ≤2 on PCAT-Complex Disability (60% capture, Rule 5).

Ten-fold cross-validation achieved 73% accuracy, with sensitivity to “not Emerged” at 78% and specificity to “Emerged” at 65%. No further rules were found.

## Discussion

In this retrospective analysis of a large national rehabilitation dataset, we applied both traditional statistical and machine learning techniques to determine whether items contained within the routinely collected UKROC clinical dataset might help to predict which patients admitted in PDOC may emerge into consciousness by the end of the programme.

Initial filtering resulted in 12 significant variables for further analysis.

Stepwise logistic regression (LR) identified a six-variable data fit model consisting of RCS-Medical, NIS-Motor, NPDS-Communication, PCAT-Behaviour, PCAT-Mood, and PCAT-Complex Disability management, which attained 71% data fit accuracy. Repeating LR with just these six variables entered as categorical (allowing selection of subsets of variable values) resulted in an improvement of variance explained to between 28% and 38%, with 77% data fit accuracy. The LR model fitted non-emergence (sensitivity 86%) better than emergence (specificity 66%). Logistic discriminant analysis identified a similarly accurate data fit model (71% accuracy). Cross-validation accuracy remained at 71%, which shows reasonable predictive capability overall on test cases, with sensitivity a high 84%. However, cross-validation specificity was poor at 52%, indicating that there was insufficient information in the training samples for reliable prediction.

An artificial neural network (ANN) data fit model produced 89% accuracy (95% sensitivity, 82% specificity) and returned an area under the ROC curve of 0.94, showing very good discriminative ability, although cross-validation reduced overall accuracy to 69% (78% sensitivity, 57% specificity). NIS-Motor, NPDS-Communication, PCAT-Behaviour, and PCAT-Complex Disability featured as the most important variables for classification accuracy (over 80% normalised importance). Given that the ANN correctly predicted 78% of patients remaining in “non-Emerged” but only 57% of patients in “Emerged” after 10% of samples were repeatedly removed in cross-validation, this drop in predicted specificity in comparison to the data fit model (82%) indicates that critical information concerning “Emergence” within patient data is being lost in these four most important variables. Rule induction produced a rule for non-emergence from PDOC with 91% sensitivity involving NIS-Motor (admission >13), PCAT-Complex Disability (admission score >2), and diagnosis subcategories of Anoxia, Stroke or Toxic. The best rule for Emergence (60%) involved NIS-Motor (admission score of 12 or 13), PCAT-Behaviour (admission ≤ 1) and PCAT-Complex Disability (admission ≤ 2). Cross-validation resulted in 73% accuracy (sensitivity 78%, specificity 65%), indicating reasonable predictive performance when reasons for classifications were required.

Triangulation through these various techniques therefore quite consistently identified characteristics that are associated with emergence from PDOC. Severe motor impairment, high need for complex disability management, medical instability and specific aetiology were predictive of non-emergence, whereas those with less severe motor impairment and agitated behaviour were predictive of emergence. These findings resonate with clinical experience. Non-emergence (78%–95% sensitivity) was modelled and predicted more accurately than emergence (57%–82%). The ANN with hidden layer produced the best model, indicating that the problem may be best addressed through non-linear techniques.

Our results compare favourably with previous machine learning approaches used in neurorehabilitation modelling. For instance, Xue et al. [18], when predicting risk of care readmission, found that logistic regression achieved 84% test accuracy in comparison to support vector machine (SVM) and random forest figures of 81% using FIM-only measures. Higher specificity figures were reported in comparison to sensitivity. Verrusio et al. [17] used Comprehensive Geriatric Assessment (CGA) measures to predict patient disability levels one year ahead. Their SVMs achieved higher predictive accuracy for patients in three classes (self-sufficiency, disability risk, and disability) than linear regression models (84% vs 67%, respectively). Zhu et al. [19] used k-nearest neighbours and SVNMs to identify older patients for rehabilitation potential and planning using Activities of Daily Living Clinical Assessment Protocol (ADLCAP) measures. High false-positive and false-negative rates were reported, providing further evidence of the difficulty in identifying suitable neurorehabilitation measures for accurate prediction.

Our results of 95% sensitivity and 82% specificity with ANNs for patients who remain in PDOC versus those who emerge (as identified by their discharge FIM + FAM scores) are therefore in line with previously reported research outcomes using machine learning approaches. One important difference in our approach is the range of multi-measurement tools used to provide data across a number of different dimensions for predicting emergence. The predictive importance of Motor measurement from the NIS questionnaire and Communication measurement from the NPDS questionnaire, as well as of a number of measures from the Patient Categorisation Tool, provides evidence that predicting outcomes of patients after they enter rehabilitation will need analytical techniques that can cater for a wide variety of measures (categorical, ordinal, scale) in an integrated manner. Finding statistical and machine learning techniques that offer this facility is likely to be the main challenge in neurorehabilitation research for the foreseeable future. Nevertheless, the induction of a highly accurate but narrow rule involving aetiology on admission provides a pointer as to where research hypotheses can be focussed in future.

### Strengths and weaknesses

Strengths of this study include the large size of the dataset, gathered in the context of real life clinical practice across over 50 centres in England, which supports the generalisability of the findings. The use of tools that are routinely collected within the national clinical dataset confers the advantage of utility as the data to inform prediction should be readily available to clinicians at least in the UK. Moreover, these tools are readily available and free to use for anyone wishing to do so in other countries.

The authors acknowledge some differences between this study population and others that have explored outcome of patients in PDOC. Firstly, this was a relatively young population (mean age 48.4 (SD 15.8) as the UK specialist rehabilitation services are generally targeted at working aged adults, and this may explain the lack of age effect that is seen in other studies. Although our range of aetiologies was similar to other studies, the proportion of traumatic brain injury (TBI) was lower than in some, e.g., Giacino and Kalmar [6]. The US study followed patients from an early acute stage, while our group did not present until an average of eight months post injury, and it is possible that many of the TBI patients had already emerged by this stage. A relative strength of our study is that it examines patents further down the line from some other published studies. We believe that our findings are likely to have reasonable generalisability for the younger adult population of patients who still present in PDOC some 6-12 months post injury.

The most significant weakness of the study is that the diagnosis of both PDOC and emergence from it is gathered by proxy from the UK FIM + FAM. While our previous paper demonstrated the sensitivity and specificity of the FIM + FAM for identifying patients in PDOC it remains to validate the FIM + FAM in this context by direct correlation with a “gold standard” such as the Coma Recovery Scale or the Wessex Head Injury Matrix. Although there is a precedent for using the FIM as a proxy for “vegetative state” and, with its additional 12 cognitive and psychosocial variables the FIM + FAM might be expected to provide a more sensitive test of cognitive interaction, we cannot be certain about the diagnosis of PDOC or emergence in this analysis. Cases in the grey zone (FIM + FAM score 32-25) were excluded to reduce the chance of cross contamination. The UKROC database is currently being expanded to include a dedicated PDOC registry. This will enable routine collation of tools specifically designed to record the level of consciousness including the Coma Recovery Scale and the Wessex Head Injury Matrix. Once this is formally established and a sufficient body of multicentre data has been collected, future ML studies may include these direct measures of consciousness to enhance the certainty of PDOC diagnosis.

On the other hand, on a pragmatic level, it is reasonable to make use of the extensive data that are available. Moreover, clinical decision-making in the context of PDOC has evolved over recent years. Decisions to give, continue or withhold active medical and life-sustaining treatments in the UK are no longer simply based on whether or not the patient will emerge into consciousness, but whether they will recover a quality of life that they themselves would value. In this context the FIM + FAM could potentially form a more useful framework for discussing the type of functional profile that an individual patient might consider acceptable—for example some people may tolerate physical disability if they had sufficient cognitive and communicative ability, while others may feel differently. Future longitudinal analyses would therefore focus not simply on emergence but the ultimate functional outcome for these catastrophically brain-injured patients.

## Conclusion

Notwithstanding these recognised limitations, this study demonstrates the potential opportunities to enhance the prediction of outcome using machine learning techniques to explore the richness of routinely collected clinical data.

## Data Availability

As the UKROC data set is a live clinical data set, for reasons of confidentiality and data protection data sharing is not available at the current time. *Copies of the tools* used in this study are available free of charge from the authors. Please visit our website for more details and contact information. http://www.kcl.ac.uk/lsm/research/divisions/cicelysaunders/research/studies/ukroc/tools.aspx
